# Graphical displays for effective reporting of evidence quality tables in research syntheses

**DOI:** 10.1186/s12978-016-0130-3

**Published:** 2016-03-09

**Authors:** Luciano Mignini, Rita Champaneria, Ekaterina Mishanina, Khalid S. Khan, with the EBM-CONNECT Collaboration

**Affiliations:** Centro Rosarino de Estudios Perinatales, Rosario, Santa Fe Argentina; Birmingham Clinical Trials Unit, School of Cancer Sciences, College of Medicine and Dentistry, University of Birmingham, Birmingham, UK; Colchester University Hospital, Colchester, UK; Centre for Health Sciences, Barts and The London School of Medicine and Dentistry, Queen Mary University of London, London, UK

**Keywords:** Grade, Grade plots, Guidelines, Radar charts, Systematic reviews, GRADE plots allow easier, Quicker and more accurate identification of deficiencies in the quality of studies, Compared to tabulated results

## Abstract

**Background:**

When generating guidelines, quality of the evidence is tabulated to capture its several domains, often using the GRADE (Grading of Recommendations Assessment, Development and Evaluation) approach. We developed a graphic display to capture deficiencies, outliers and similarities across comparisons contained in GRADE tables.

**Methods:**

Based on a systematic literature review capturing the effects of 32 different therapeutic comparisons on dysmenorrhoea, we synthesised evidence quality in tables and graphs. We evaluated time taken to accurately assess evident quality and preference for tables vs graphs.

**Results:**

The plots provided visually striking displays of strengths and weaknesses of the evidence across the spectrum of comparisons on a single page. Equivalent tabulated information spread over 4 pages. Participants preferred and interpreted graphs quicker and more accurately than tables.

**Conclusions:**

The graphic approach we developed makes interpreting evidence easier. Large tables are dry and cumbersome to read and assimilate. When guideline statements are accompanied by these plots, they have the scope for improving the credibility of the recommendations made, as the strength of the evidence used can be clearly seen. Further empirical research will establish the place for graphic displays.

## Abstract (es)

**Resumen:**

Para generar guías de práctica clínica, la calidad de la evidencia se tabula, a menudo utilizando el GRADE (Clasificación de las recomendaciones de la evaluación y desarrollo). Hemos desarrollado una pantalla gráfica para capturar deficiencias, valores atípicos y similitudes a través de comparaciones que figuran en las tablas GRADE.

**Métodos:**

Basado en una revisión sistemática de la literatura para analizar los efectos terapéuticos de 32 diferentes tratamientos en la dismenorrea, se sintetizó calidad de la evidencia en tablas y gráficos. Evaluamos tiempo necesario para evaluar con precisión evidente calidad y preferencia por las tablas vs gráficos.

**Resultados:**

Los gráficos muestran fácilmente las fortalezas y debilidades de la evidencia en todo el espectro de las comparaciones en una sola página. Los participantes prefieren los gráficos y estos son interpretados más rápido y con más precisión que las tablas.

**Conclusiones:**

El enfoque gráfico que hemos desarrollado hace más fácil la interpretación de la evidencia. Las tablas son difíciles y engorrosas para leer y asimilar. Cuando los estados únicamente se acompañan de estos gráficos, tienen la posibilidad de mejorar la credibilidad de las recomendaciones, ya que la fuerza de la evidencia utilizada se puede ver claramente. Más investigación empírica establecerá el lugar para pantallas gráficas.

## Background

When using scientific evidence for clinical decision making, it is essential to know its quality, in order to be confident in the recommendations [18]. The Grading of Recommendations, Assessment, Development and Evaluation (GRADE) is a quality assessment tool used to evaluate limitations of the evidence and to provide an underpinning strength to recommendations [1]. GRADE tables describe various quality parameters including study design, risk of bias, inconsistencies, indirectness and imprecision, to generate an overall rating of the evidence (from high to very low). To be comprehensive, the quality parameters (criteria, factors or domains) have to be included for each outcome and comparison separately, making the tables bulky and difficult to use [3, 20]. These could be presented as graphic displays compressing a large amount of data into concise, easy to interpret figures [12]. This article explores how addition of graphic displays of evidence quality assessment to GRADE may help readers, providing the findings of a user evaluation.

## Methods

### Tabulating evidence profiles

Primary dysmenorrhoea, a common idiopathic chronic pelvic pain syndrome of unknown aetiology [13], was chosen as an example. We used this topic to demonstrate the difficulties encountered when bringing together complex data on quality of the evidence on numerous comparisons. In this systematic review we searched electronic literature databases, including Medline, Embase and the Cochrane Library until January 2010 [14]. Harms alerts from relevant organizations such as the US Food and Drug Administration (FDA) and the UK Medicines and Healthcare products Regulatory Agency (MHRA) were also searched. We selected randomised controlled trials (RCTs) which were at least single blinded, with at least 80 % follow up at primary end point, and had a sample size of at least 10 women in each group.

There were many interventions compared for effect on various outcome measures. For each comparison and outcome pair, evidence was initially graded by the study design. We assigned all evidence a high level of quality as it was based on a RCT design. If there were deficiencies in the domains risk of bias, inconsistency, indirectness and effect size or its precision, the quality level was downgraded by one level (if the deficiency was classified as serious) or by two levels (if the deficiency was classified as very serious). An example of an evidence profile is shown in Table 1. The full tabulation of the evidence profile spread over 4 pages (over 1,500 words).

**Table 1:** The features of evidence grading captured in a GRADE plot (adapted from Evid Based Med 2011;16:65-9)

Grade	Design	Risk of bias	Inconsistency	Indirectness	Effect size	Evidence Quality
	Studies are either described as randomised-control trials (RCTs) or observational.	Explains the limitations of the study based on assessment of blinding and allocation process, follow-up and withdrawals, scarcity of data, other methodological concerns e.g. incomplete reporting, subjective outcomes.	Inconsistencies due to unexplained (statistical) heterogeneity. The same weakness is only downgraded once.	Presence of indirectness in the PICO elements that affect the generalisability of participants and outcomes from each study to population of interest.	Relates to imprecision of the estimated effect based on the reported odds ratios or relative risks or mean differences for comparison. This is based on the confidence intervals, sample size and number of events.	
High	Randomised controlled trial	No problems	All/most studies show similar results with or inconsistency across studies is explained by a dose response	Population and outcomes broadly generalisable	Effect size more than 5 or less than 0.2 for all studies/meta-analyses included in comparison and significant	
Moderate			Lack of agreement between studies (e.g. statistical heterogeneity between RCTs, conflicting results)		Effect size more than 2 or less than 0.5 for all studies/meta-analyses included in comparison and significant	
Low/Very low	Controlled observational study	Problem with 2 or more elements	Serious lack of agreement between studies	Some problem with 2 or more elements	Not all effect sizes more than 2 or less than 0.5 and significant; or if effects observed not significant	
Example^a^: Thiamine vs Placebo for Pelvic Pain	Randomised trial	No limitations	Consistent	Indirect	Precise	Moderate
	Initially assigned a high strength level	→ No Change	→ No change	→ Relegation	→ No change	┘

^a^based on evidence profile shown in Fig. 1 and BMJ 2012;344:e3011 doi:10.1136/bmj.e3011

### Graphically displaying evidence profiles

Radar charts were used to summarise data concerning several variables in a two-dimensional graph. Each chart is made up of a number of spokes or radii, each representing a variable, arranged at equal angles. Three or more quantitative variables can be represented in this way for summarising quality parameters of clinical evidence.

A radar chart, or GRADE plot, consisting of the five most important GRADE quality parameters (study design, risk of bias, inconsistency, indirectness and imprecision of effect size) was created for each comparison and clinical outcome. The length of a spoke was proportional to the magnitude of the quality of that parameter, ranging from serious deficiency (no spoke) to no deficiency (full spoke) (Table 1) [9, 12]. If all quality parameters were of high magnitude, a GRADE plot will be of symmetrical pentagon shape. However, if one of the parameters was deficient, the shape of the pentagon will be distorted and the cross-sectional area will be smaller. GRADE plots were constructed based on a variety of treatments for pelvic pain.

Overall quality of the evidence is rated by GRADE using the following categories, high, moderate, low and very low. We utilised a traffic light colour coding scheme to represent this grading scale on GRADE plots. The following colours were allocated for each rating: green for high quality evidence, yellow for moderate, red for low/ very low and white where evidence had no quality rating available. Colour coding of the data used to construct the GRADE plots and the area covered within the plot provided additional visual information regarding the quality of the evidence rating. Data for GRADE plots were double checked to avoid error.

## Results

### Quality of evidence on pelvic pain

**Fig. 1:**
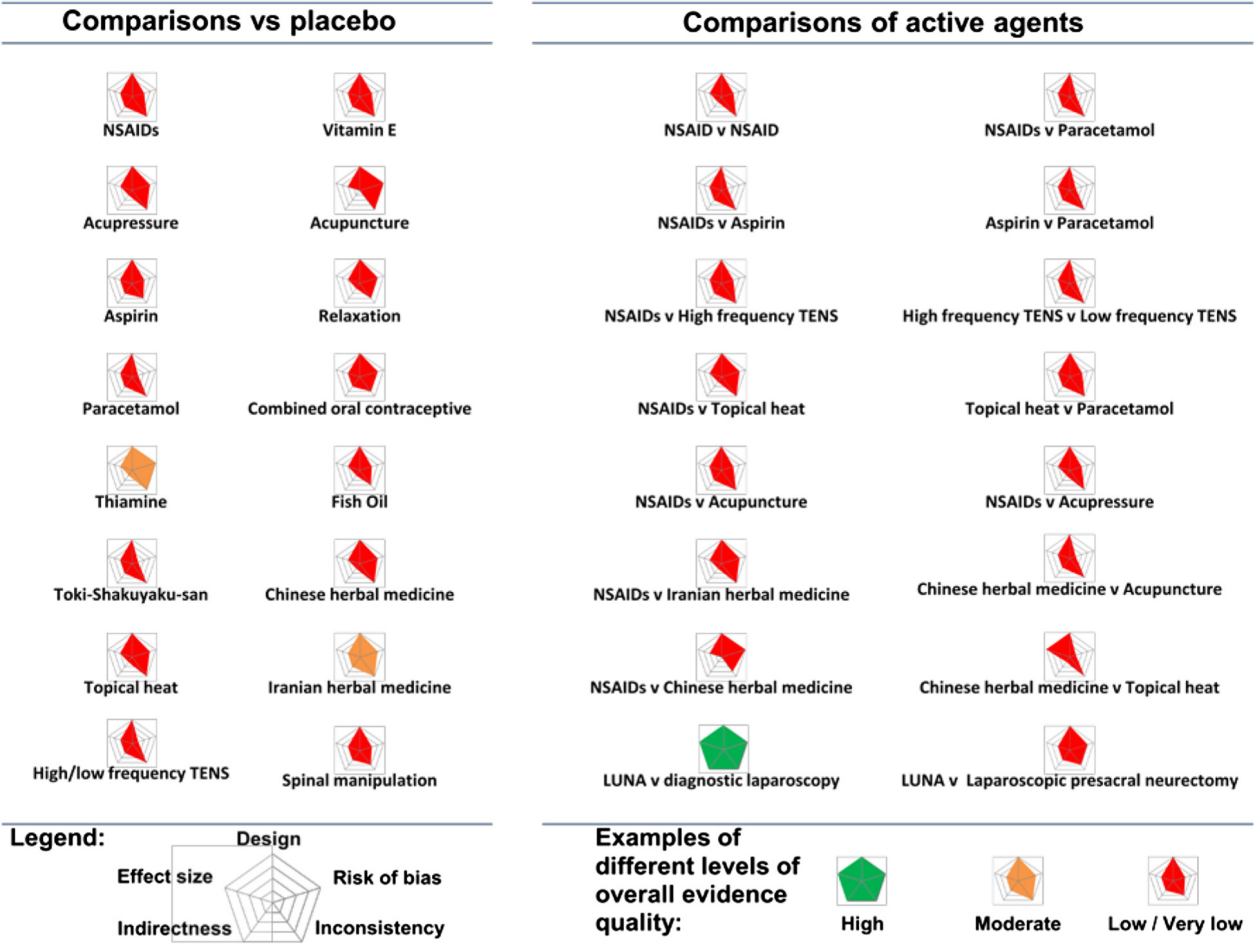
Effect of various treatments on pelvic pain: Graphic overview of evidence quality. *Each graph represents the quality domains shown on concentric spokes. Starting from 12 o’clock and moving clockwise these are design, risk of bias, inconsistency of results, indirectness of participants, and outcomes and effect size* [12]*. For each of the spokes, the length represents the magnitude of quality adapting the scoring system used for Clinical Evidence reviews (* http://clinicalevidence.bmj.com/x/set/static/ebm/learn/665072.html *). The block shapes, formed by joining the lengths of the spokes, colour coded to represent the overall quality of evidence as follows: Green = high quality evidence; yellow = moderate quality; and red = low or very low quality. See main text of our Clinical Evidence review* [14] for details. NSAIDs = non steroidal anti inflammatory drugs TENS = transcutaneous electric nerve stimulation LUNA = laparoscopic uterine nerve ablation. * The lack of a clinically meaningful effect of LUNA for dysmenorrhoea has been confirmed through an individual patient data meta-analysis [6]

Figure 1 shows the quality of the evidence for agents commonly used to treat dysmenorrhoea. When comparing single agents against placebo or no treatment, we found that the quality of the evidence ranged from moderate to low/very low. Most of the comparisons assessed had a high risk of bias and none of them directly compared interventions which we were interested in or measured outcomes important to patients. Moreover, none of comparisons had an adequately large and precise effect size.

Head to head comparisons of different active agents for the treatment of pelvic pain highlighted many gaps in the evidence base. Deficiencies in quality were seen in the majority of data plotted (Fig. 1). There was only one high quality comparison (LUNA vs. diagnostic laparoscopy) that complied with all the quality items assessed. All the remaining comparisons were of low/very low quality. Non steroidal anti inflammatory drugs (NSAIDs) [2, 7, 8, 15, 19] and hormonal regulation through oral contraceptive pills [22] were significantly more effective for pain relief than placebo. Despite poor quality these interventions remain in common use [5, 21]. Furthermore, there was severe risk of bias and all but one comparison showed an inadequate effect size.

The GRADE plots immediately captured evidence quality, for example, there was moderate quality (yellow light) evidence of the effectiveness of Thiamine and Iranian herbal medicine for treating dysmenorrhoea and for the others treatment therapies there were low to very low quality evidence GRADE plots depicted in red. When therapies were compared head to head, there was only one high quality comparison (green light), which suggested that LUNA (laparoscopic uterosacral nerve ablation) should not be undertaken as a treatment for dysmenorrhoea as surgery was not found superior to no intervention. Other comparisons provided weaker recommendations, again also highlighted in red.

### An empirical evaluation comparing tables vs graphs

We conducted a small randomised evaluative study to determine whether researchers and clinicians interpreted graphs quicker and more accurately than tables. Their preference for one or the other of the two approaches was also assessed. Prior to randomisation participants were shown a powerpoint presentation which explained the GRADE quality assessment tool and the new graphic concept. Seventeen participants (7 researchers and 10 hospital doctors) were then randomly assigned to either the intervention group (graphs) or the control group (tables). Participants in the intervention group were presented with a summary figure of 10 graphs summarising 5 quality parameters (study design, risk of bias, inconsistency, indirectness and imprecision). The overall quality of the evidence was indicated with the aid of colours (green for high overall quality, yellow for moderate and red for low and very low). The participants were then asked 10 questions regarding the quality of the evidence. Participants in the control group were presented with the same information and asked the same questions, but this time summarised in tables. The time taken to complete the questionnaire was recorded in seconds with the aid of a stopwatch. The preference between graphs or tables was then recorded.

**Fig. 2:**
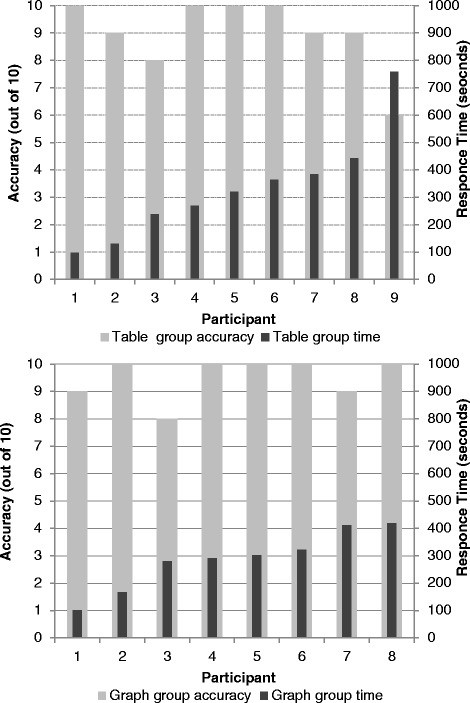
Comparison of accuracy and time taken to interpret equivalent Grade tables (*above*) and graphs (*below*)

We found that on average graphic displays were interpreted quicker and more accurately than tables (see Fig. 2), however the numbers of participants were too small to draw any statistically significant inferences. The majority of the participants preferred graphs to tables. Among those who received graphs 7/9 indicated a preference for graphs vs 6/8 amongst those who received tables.

## Discussion

Although the GRADE quality assessment tool is not used universally, it has been utilised by some large guideline producing bodies including WHO (World Health Organization) and NICE (National Institute for Clinical Excellence). Currently, the summaries of GRADE quality assessments are presented in lengthy tables that often slow the reader down in interpretation of the findings. GRADE plots can summarise quality assessment in a more concise explicable way, making it quicker to decide on the value of the evidence. It is also possible to arrange the results of multiple interventions and outcomes in a compressed manner that can be easily examined and compared. The quality is further made explicit in the graphs by use of colour-coding, which is a strength of this approach.

There has been empirical research comparing tables and graphs of equivalent data. The compositional format and content of quantitative data displays has an impact on people’s comprehension, choice and preference. [11] Our evaluative study showed that participants preferred and interpreted graphs quicker and more accurately than tables. One deficiency of our work is that we did not cover every single aspect of the GRADE approach. We also modified some aspects of GRADE to create this exemplar. For further development aspects such as publication bias and criteria for upgrading or downgrading will need to be additionally considered, while strictly adhering to the GRADE system. Another consideration should be the balance of benefit vs risk of harm. It is important to remember that the underlying concept behind the graphs is to visualise the GRADEing for the ease of assimilation by users, not to replace the in-depth analysis and consideration necessary for formulation of recommendations. Further, it is necessary to recognise the pilot or preliminary nature of our empirical evaluation. Stronger empirical work will be required to advance the advantages of graphs that show potential in our work.

Brewer et al. found that patients needed to see bar charts for a shorter amount of time compared to tables to understand the same results [4]. Bauer et al. also concluded that physicians worked significantly faster with the graphical display than tables [3]. The overall quality of the evidence can be colour coded with a traffic light system also used to display health economic data. [17]. The use of colour is not only eye-catching but if used appropriately can allow the reader to capture the overall quality immediately [11]. This idea is supported by the results of a trial by Hawley et al. who found that colour graphical representation of results (pictographs) were the most effective way of conveying information [10]. McCaffery et al. agree with these findings. Their trial reported that in adults with lower education and literacy, pictographs were the best format for displaying numerators of less than 100 (<100/1000), and bar charts were best for larger numerators (>100/1000) [16]. We therefore suggest that this strength of evidence and resulting recommendations could easily be demonstrated with a colour-coded system.

## Conclusion

GRADE plots can be used to summarise large amounts of data in a concise, easy to interpret way. They demonstrate the quality parameters of study design, risk of bias, indirectness, inconsistencies and imprecision. The colour coded cross sectional area of the pentagon represents the overall quality of the evidence, also highlighting the strength of the recommendation. These plots provide a useful means of visually displaying evidence that could be adopted alongside the GRADE approach. The summary obtained through the plots can be read at a glance to immediately identify deficient areas that can be explored further with GRADE tables. Based on our findings, we would like to suggest to guideline makers to use graphic displays when summarising and publishing conclusions on multiple comparisons and outcomes.
